# Sucrose, Lactose, Thermogravimetry, and Differential Thermal Analysis: The Estimation of the Moisture Bond Types in Lactose-Containing Ingredients for Confectionery Products with Reduced Glycemic Index

**DOI:** 10.1155/2023/8835418

**Published:** 2023-09-05

**Authors:** Elena Melnikova, Ekaterina Bogdanova, Daria Paveleva, Igor Saranov

**Affiliations:** Voronezh State University of Engineering Technologies, Russia

## Abstract

This research is aimed at conducting a comparative analysis of the dehydration process of lactose-containing ingredients and sucrose. Differential scanning calorimetry (DSC) and thermogravimetry (TG) methods were used to investigate the moisture bond types and the process of thermal degradation in native whey, dried skimmed milk, dried whole milk, dried milk whey demineralized, purified lactose, and sucrose. There were several peaks on the DSC and TG curves for lactose-containing ingredients. They determine the loss of the physically absorbed water on surfaces, physically occluded water and hydrate-forming water (up to 180°C), anomerization of lactose (160–220°C), and melting followed by decomposition (above 230°C). The multiple peaks on the dDSC curves from 135 to 170°C indicate the course of the Maillard reaction in the mix with proteins. For the native whey, the amount of chemically bound water was 56.11 ± 1%; for the dried whole milk, it was 59.86 ± 1%; for the milk whey demineralized, it was 64.56 ± 1%; and for the dried skimmed milk, it was67.17 ± 1%. The results are presented as mean values ± SD (*n* = 3) and were considered statistically significant when *P* < 0.05. The similar to some extent form of the DSC and TG curves to lactose-containing ingredients and granulated sugar confirms the possibility of using them as the component of bakery and confectionery product formulas to reduce the glycemic index and improve organoleptic properties of products as well as correct their mineral compositions. Considering the constant changing of technologies for obtaining various dairy ingredients as well as the occurrence of their new kinds, such studies contribute to the expansion of existing knowledge and reference data.

## 1. Introduction

Granulated sugar and its main component sucrose (up to 99.98%) are one of the most widespread products of high demand in bakery and confectionery product manufacturing.

Sugar consumption physiological standards recommended by the Ministry of Health of the Russian Federation should be 24 kg per person per year (sugar-containing products are included) (Methodological recommendations MR 2.3.1.0253-21, 2021) [1]. The statistics show that according to the 2022 results, the Russians' sugar consumption will be considerably exceeded and may amount to more than 31 kg per person. However, according to WHO guidelines, excessive intake of free sugars (monosaccharides and disaccharides) causes body weight gain and dental caries. Therefore, it is recommended to reduce their consumption to less than 10% of the total energy intake (guideline of [2]). Moreover, all carbohydrates are characterized by a certain glycemic index which is determined by the level of postfood glycemia due to their consumption relative to glucose. This indicator characterizes the duration of satiety, the frequency and amount of food intake, the load on the pancreas because of the need to produce insulin, and the regulation of body weight [3]. Carbohydrates with a high glycemic index (such as sucrose, GI 65; glucose, GI 100; maltodextrins, GI 103; and maltose, GI 105) [4, 5] increase the blood glucose level, which decreases only with active physical exercises. The production of a significant amount of insulin is required for the absorption of such carbohydrates. Аn insulin response does not occur in the human body after consumption of carbohydrates with a glycemic index less than 55 (such as isomaltulose, GI 2; fructose, GI 23; and lactose, GI 46) [4, 5].

The excessive consumption of sucrose, characterized by the high glycemic index, results in diseases development such as obesity, diabetes, cardiovascular morbidity, liver and kidney disorder, and consequently leads to lifetime reduction. The adjustment of food patterns belongs to one of the strategies of such problem decision, in particular the decrease in product consumption with the high glycemic index or the partial substitution of sucrose with other carbohydrates and different ingredients [6, 7].

The dried skimmed milk (GI 45), dried whole milk (GI 32), dried milk whey (GI 43), and dried permeate (GI 41) may be referred to such ingredients; their main component is carbohydrate of the animal origin, lactose, which is characterized by glycemic index of 46 and sweetness coefficient of 0.4 regarding sucrose [8].

The prognosis of lactose and sucrose thermal behavior is of high importance for these ingredients used in the technology of confectionery product manufacturing. It provides the heat treatment of sugar-containing products. The thermodynamic properties of food ingredients specify the drying period, mixing behavior, packaging choice, moisture change, and storage stability. The variation of some thermodynamic properties with regard to moisture content defines the sorption mechanisms at all stages of the technological process.

Various methods can be used to determine the types of moisture in different systems. For example, they are drying of the sample at a constant temperature, Karl Fischer titration, drying at infrared radiation, calorimetry, and thermogravimetry. The last one is more accurate and objective and has been used by several researchers to analyze the types of moisture in various foods [9–16].

Differential scanning calorimetry (DSC) and thermogravimetry (TG) allow to study thermodynamic characteristics and the ratio of free and bound moisture to evaluate the applicability of lactose-containing ingredients in bakery and confectionery products with reduced glycemic index.

The research is aimed at conducting a comparative analysis of the dehydration process of lactose-containing ingredients and sucrose. Considering the constant changing of technologies for obtaining various dairy ingredients as well as the occurrence of their new kinds, such studies contribute to the expansion of existing knowledge and reference data. Besides, lactose is characterized by its ability to mutarotate in aqueous solutions, hygroscopicity, and a more complex crystal lattice compared with sucrose. This may cause differences in the functional and technological properties of lactose-containing ingredients relative to sucrose in the same food systems and perhaps will require adjustments to a few technological modes in the case of sucrose replacement. In the furtherance of this goal, the following tasks have been defined:

- to study thermodynamic properties and the ratio of free and bound moisture in lactose-containing-based materials and sucrose
- to elaborate the technological recommendations for partial substitution of sucrose with different lactose-containing ingredients in confectionery product formulas

## 2. Materials and Methods

### 2.1. Objects

The white beet sugar crystallized (granulated sugar), produced according to GOST 33222-2015 (Russian Federal Standard); purified lactose (GOST 33567-2015); dried skimmed cow milk with dry fat matter (FDM) not more than 1.5% *w*/*w* and dried whole cow milk with FDM not less than 26.0% *w*/*w*, produced in accordance with GOST 33629-2015; dried cow milk whey demineralized (GOST R 56833-2015); and dried whey product (native whey obtained from skimmed cow milk as an experimental sample after isolation of micellar casein concentrate), produced at PJSCDP “Voronezhskii” (“Kalacheevskii Cheese Factory”, Kalach, Voronezh oblast). These samples were stored at 75% relative humidity at 25°C in closed retail packages.

### 2.2. Methods

Experimental studies were carried out according to generally accepted and arbitration methods in the Research Equipment Sharing Center “Control and Management of Energy-Efficient Projects” at FSBEI HE “Voronezh State University of Engineering Technologies”.

The moisture content of the samples was determined by their heating to 105°C according to GOST 29246-91; total protein content in dry matter was studied using the Kjeldahl method according to GOST 34454-2018 fat content in dry matter was determined by the gravimetric method according to GOST ISO 1736-2014; ash content in dry matter was studied using the dry combustion method according to GOST 34845-2022; and lactose content was calculated from the difference between the dry matter and other components. The content of lactose anomeric forms in the samples was determined using polarimeter KRUSS P1000-LED (Germany) by polarimetry [17].

The experiments were carried out at a simultaneous thermal analysis unit, the model STA 449 F3 Jupiter (Germany); the heating temperature varied from 26 to 320°C with the speed of 5 K/min in the oxidized aluminum pans; the sweeping gas was nitrogen at a rate of 60 cm^3^/min. Calibration substances (C10H16, C12H10, In, Bi, and Zn) were previously measured in a type S carbide-silicon furnace with the heating speed of 5 K/min in the oxidized aluminum pans to calibrate the temperature and sensitivity of the type S sample holder; the sweeping gas was nitrogen at a rate of 60 cm^3^/min. The obtained temperature and enthalpy values were used to calculate and create calibrations using the programs Temperature Calibration and Sensitivity Calibration (Germany). The temperature and loss of weight measurements during the dehydration and sample decomposition processes were defined by DSC and TG. The obtained data were investigated with the application of computer software NETZSCH Proteus and MS Excel in order to construct the derived curves dDSC and dTG. The conversion degree *α* for each sample was calculated as the ratio of mass at a certain time to the total mass change at the end of the process according to TG curve data.

The content of 5-hydroxymethylfurfural was determined by the Winkler photometric method [18] using a PE 5400 UV spectrophotometer (Russia). The absorbency of the samples was measured at a wavelength of 550 nm in optical cells with a working length of 30 mm [19, 20]. The content of 5-hydroxymethylfurfural (HMF), mg/kg, was calculated according to equation:

(1) $$\begin{array}{c} \text{HMF}=\frac{192∙\text{absorbance}}{\text{cuvette thickness }\left(\text{cm}\right)}, \end{array}$$

where 192 is the dilution factor.

Browning index was calculated as the difference between two supernatant absorbances (at 420 and 600 nm) after precipitation and centrifugation of samples [21].

### 2.3. Statistical Analysis

The samples were investigated 5–10 times in triple sequence. The results processing, diagram construction, and their description were made with the help of mathematical statistics methods of Microsoft Office 2021 for Mac application. The results are presented as mean values ± SD (*n* = 3) and were considered statistically significant when *P* < 0.05.

## 3. Results

**Figure 1:**
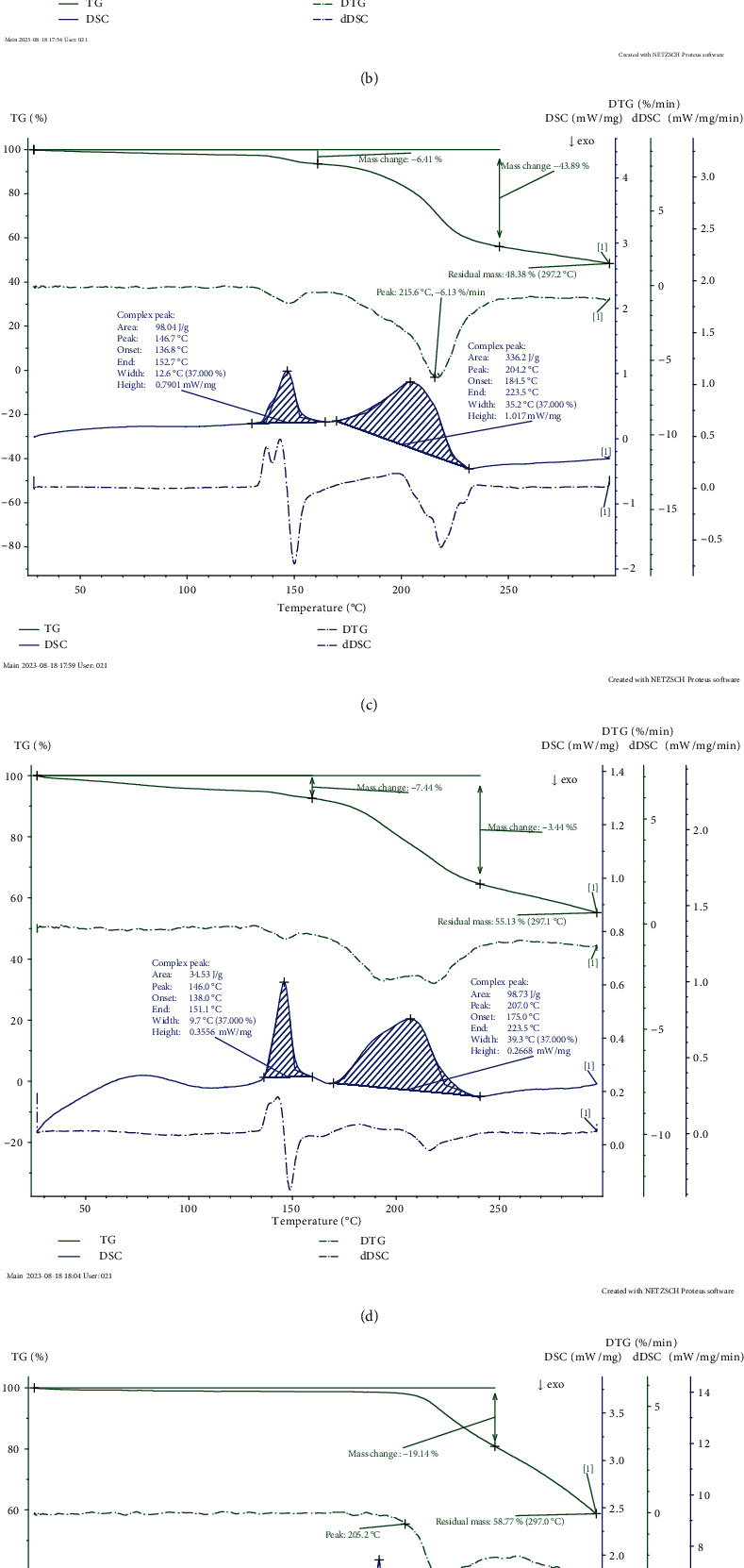
Thermograms of the tested samples. (a) Dried skimmed milk. (b) Dried whole milk. (c) Dried native whey. (d) Dried milk whey demineralized. (e) Granulated sugar. (f) Purified lactose.

The thermogram types are presented by specific curves (Figure 1). It is worth noting that different components and agglomerates affect the type of these thermograms; however, the lactose as the main component of these products determines their bends [22]. It was found lactose presence in the crystalline form predominantly with the simultaneous presence of a certain ratio of the anomeric forms in the studied samples of dairy products (Table 1).

The smooth curve bend in the temperature range from 40 to 120°C is caused by the results of the evaporation of surface water and trapped in pores and capillaries from the analyzed samples. The moisture content (Table 1) shows the amount of water physically absorbed on the surfaces. The first endothermic peaks at a temperature of about 135°C are monohydrate desorption or refer to the removal of hydrate-forming water, accompanied by the heat absorption and mass change of the samples on the TG curves.

The dDSC peak area is proportional to the enthalpy change of the reaction and to the sample mass change (Table 2) and inversely proportional to their thermal diffusivity. The enthalpy characterizes energy changes that occur in food ingredients at different stages of hydration caused by differential alternations of moisture equilibrium [23].

The high index of enthalpy for native whey compared with the tested ingredients may be caused by the grade of moisture binding with native forms of components and specific correlation of protein and lactose; mass change (43.89%) may indicate the maximum amount of the removed bound moisture of the 2nd stage of dehydration [24].

**Figure 2:**
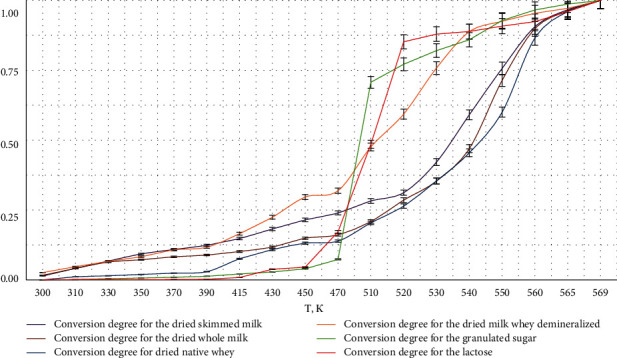
The dependence of substance *α* conversion on the temperature.

Figure 2 shows the dependence of conversion degree *α* on the temperature *T* and *K* calculated according to the TG curves for several temperature intervals. The S-shape indicates the three-step process of dehydration with different speed of water release and its hard interaction with lactose, whey proteins, and other components of the tested dairy ingredient samples. The difference in the conversion degree curve for granulated sugar and purified lactose is correlated with the chemical composition and the hydrogen bonds breaking before melting of the crystal structure, which encloses the chemically bound water molecules [25, 26].

Stage I is the section where the removal of physically absorbed on surfaces and physically occluded water restrained by the weak capillary forces were detected. Stage II is the section where hydrate-forming water was removed (water molecules, forming more distant adsorption layers, and crystallohydrates). Stage III is the section where chemically bound water, which characterizes monomolecular water layer bound with polar groups and water-dipole interactions, is removed; it corresponded to the residual moisture after drying the weight quantity (Table 3).

### 3.1. Discussion

Three peaks can be clearly identified on the DSC curve of lactose (Figure 1(f)). They determine loss of the crystallization water (90–180°C), anomerization of the *α*-form of lactose to the *β*-form (182–235°C), and melting followed by decomposition (235–263°C). This corresponds to the results obtained by other researchers [27–29].

The summating DSC curve for the granulated sugar indicates two endothermic peaks with maximum values 192°C and 222°C accordingly. Removing water from sucrose crystals starts at 162°C; the melting temperature may be considered the beginning of the big peak (205°C roughly at the heating speed of 5 K/min). The visual process of sucrose dehydration (the loss of weight in derivate curve) began at 185-187°C and continued up to the finite temperature with a total loss of weight of 41.23%; the weight loss was minimal in the melting area (up to 2%) [16, 23, 24, 30].

The presence of multiple peaks on the dDSC curves from 135 to 170°C for lactose-containing ingredients (Figures 1(a)–1(d)) indicates the possible course of the Maillard reaction in the mix with proteins simultaneously with the loss of the crystallization water by lactose. The resulting derivatives provide browning of food products during baking and frying and reduce their nutritional and biological value. Therefore, the determination of the Maillard reaction temperature range in lactose-containing ingredients is important in the context of the set tasks. Since one of the intermediate products of the Maillard reaction is 5-hydroxymethylfurfural, its presence in the samples was determined after reaching the temperature of the effects as an indicator of the Maillard reaction onset to objectively isolate nonenzymatic browning on the DSC curves (Table 4). However, its reaction rate is low which is due to the low mass fraction of moisture and water activity in the studied samples.

Small exothermic peaks without mass loss by samples of lactose-containing ingredients on the dDSC curves in the temperature range 165–190°C (Figures 1(a)–1(d)) probably can characterize the transformation of the amorphous form of lactose into a crystalline form, which is consistent with the known sources [31–33]. Endothermic peaks at a temperature of about 205–225°C without noticeable weight loss may refer to the anomerization of the *α*-form of lactose to the *β*-form, followed by the melting and decomposition of the components. The rapid decrease in the mass of lactose-containing ingredients was observed with a temperature interval of 215–245°C; this may be caused by the degradation of the byproduct elementary materials.

The similar to some extent form of the DSC and TG curves to lactose-containing ingredients and granulated sugar allows to conclude that they can be used as the component of bakery and confectionery product formulas to reduce the glycemic index and improve organoleptic properties of finished products.

In bakery, the glycation process results in the taste, texture, and colour change of the finished products. During the heat treatment, the chemical transformations of the lactose-containing ingredients which are sensitive to Maillard reaction result in a high concentration of melanoidins in the finished product, and as a result, the brownie colour with the caramel (burnt sugar) flavor [34–38].

The main limitation of the conducted experimental studies is the simultaneous presence of lactose in dry dairy products in amorphous form and in the form of crystallohydrates as well as a high mass fraction of protein in them, which determines the possible course of Maillard reaction and affects the validity and interpretation of the obtained data.

## 4. Conclusion

The specific features of the chemical composition of the tested samples have determined the different ratios of moisture. For the native whey, the amount of chemically bound water was 56.11 ± 1%, and it was close in value to the dried whole milk at 59.86 ± 1%, as well as for the milk whey demineralized at 64.56 ± 1% and for the dried skimmed milk at 67.17 ± 1%. This will allow to make the partial replacement of sucrose with lactose of dairy components in confectionery formulations without any additional technological operations and to have a positive effect on the taste, colour, and glycemic load of the finished confectionary products, as well as to correct their mineral compositions. The limiting factor of such a replacement is the lower sweetness of lactose (1.0 SES for sucrose and 0.16–0.4 SES for lactose [39]) and the thermal stability of lactose-containing products (decomposition of sucrose occurs at 210–260°C, and for the studied dairy products, the end of decomposition occurs at 215-245°C). Further research will focus on the link between dataset from the present paper and physicochemical properties, and the sweetness coefficients of the studied samples.

## Figures and Tables

**Table 1:** Chemical composition of lactose-containing products.

Name of the indicator	Dried whole milk	Dried skimmed milk	Dried native whey	Dried milk whey demineralized	Purified lactose
Moisture content, % *w*/*w*	4.0 ± 0.2	4.5 ± 0.2	4.0 ± 0.3	4.0 ± 0.3	1.1 ± 0.15
Lactose in dry matter, % *w*/*w*	32.2 ± 0.7	53.0 ± 0.7	82.0 ± 0.7	80.5 ± 0.7	98.5 ± 0.7
Including *β*-form, % of the total lactose content	18.6 ± 0.7	9.3 ± 0.7	12.8 ± 0.7	15, 5 ± 0.7	2.0 ± 0.7
Total protein in dry matter, % *w*/*w*	32.8 ± 0.22	33.0 ± 0.22	9.6 ± 0.30	12.5 ± 0.30	0.1 ± 0.04
Fat in dry matter, % *w*/*w*	25.5 ± 0.20	1.5 ± 0.10	—	1.0 ± 0.10	—
Ash in dry matter, % *w*/*w*	5.5 ± 0.15	8.0 ± 0.15	4.4 ± 0.15	2.0 ± 0.15	0.3 ± 0.05

**Table 2:** Enthalpy and mass change in the process of heating of the tested samples.

Sample title	Temperature interval *Δ*Т, °С (curve DSC)	Enthalpy *Δ*Н, J/g of sample (curve DSC)		Sample mass change, %, determined by removing water from the sample in the specified temperature interval (curve TG)
		Peak 1 (refers to physically absorbed on surfaces and physically occluded water)	Peak 2 (refers to hydrate-forming water)	
Dried skimmed milk	(140–200) ± 1.5%	4.80 ± 3%	45.88 ± 3%	32.83 ± 1%
Dried whole milk	(140–210) ± 1.5%	11.99 ± 3%	113.60 ± 3%	40.24 ± 1%
Dried native whey	(135–225) ± 1.5%	98.04 ± 3%	336.20 ± 3%	43.89 ± 1%
Dried milk whey demineralized	(135–225) ± 1.5%	34.53 ± 3%	98.73 ± 3%	35.44 ± 1%
Granulated sugar	(185–244) ± 1.5%	122.10 ± 3%	139.10 ± 3%	19.14 ± 1%
Purified lactose	(162–235) ± 1.5%	191.05 ± 3%	322.17 ± 3%	19.87 ± 1%

**Table 3:** Quantity characteristics of the kinetically unequal moisture in the tested samples.

Dehydration stage	*Δ*Т, К	*Δ*t, °C	*Δα*	Mass content of the moisture removed, %
Dried skimmed milk				
I	(299 – 416) ±1.5%	(26–143) ± 1.5%	(0–0.148) ± 3%	8.11 ± 1%
II	(416 – 482) ±1.5%	(143–209) ± 1.5%	(0.148–0.250) ± 3%	24.72 ± 1%
III	(482 – 569) ±1.5%	(209–296) ± 1.5%	(0.250–1.000) ± 3%	67.17 ± 1%
Dried whole milk				
I	(299 – 413) ±1.5%	(26–140) ± 1.5%	(0–0.105) ± 3%	7.80 ± 1%
II	(413 – 481) ±1.5%	(140–208) ± 1.5%	(0.105–0.170) ± 3%	32.34 ± 1%
III	(481 – 569) ±1.5%	(208–296) ± 1.5%	(0.170–1.000) ± 3%	59.86 ± 1%
Dried native whey				
I	(299–410) ± 1.5%	(26–137) ± 1.5%	(0–0.073) ± 3%	6.41 ± 1%
II	(410–497) ± 1.5%	(137–224) ± 1.5%	(0.073–0.172) ± 3%	37.48 ± 1%
III	(497–569) ± 1.5%	(224–296) ± 1.5%	(0.172–1.000) ± 3%	56.11 ± 1%
Dried milk whey demineralized				
I	(299–411) ± 1.5%	(26–138) ± 1.5%	(0–0.148) ± 3%	7.44 ± 1%
II	(411–497) ± 1.5%	(138–224) ± 1.5%	(0.162–0.376) ± 3%	28.00 ± 1%
III	(497–569) ± 1.5%	(224–296) ± 1.5%	(0.376–1.000) ± 3%	64.56 ± 1%
Granulated sugar				
I	(299–460) ± 1.5%	(26–187) ± 1.5%	(0–0.048) ± 3%	1.0 ± 1%
II	(460–517) ± 1.5%	(187–244) ± 1.5%	(0.048–0.756) ± 3%	18.14 ± 1%
III	(517–569) ± 1.5%	(244–296) ± 1.5%	(0.756–1.000) ± 3%	80.86 ± 1%
Purified lactose				
I	(299–464) ± 1.5%	(26–191) ± 1.5%	(0–0.034) ± 3%	1.1 ± 1%
II	(464–503) ± 1.5%	(191–230) ± 1.5%	(0.034–0.856) ± 3%	18.87 ± 1%
III	(503–553) ± 1.5%	(230–280) ± 1.5%	(0.856–1.000) ± 3%	80.13 ± 1%

**Table 4:** The content of 5-hydroxymethylfurfural in experimental samples.

Sample title	Peak termination temperature on the DSC curve, °C	The content of 5-hydroxymethylfurfural, mg/kg	Browning index
Dried skimmed milk	153	5.4 ± 0.4	0.09 ± 0.02
Dried whole milk	134	20.6 ± 0.9	0.31 ± 0.02
Dried native whey	132	12.3 ± 0.5	0.17 ± 0.02
Dried milk whey demineralized	136	17.8 ± 1.1	0.024 ± 0.02

## Data Availability

The data used to support the findings of this study are available from the corresponding author upon request.

## Additional Points

*Practical Application*. This study will allow the partial replacement of sucrose by the native whey or other dairy ingredients without any additional technological operations, and to have a positive effect on the taste, colour, and glycemic load of the finished confectionary products, as well as to correct their mineral compositions.
